# Draft Genome Sequence of *Sphingobium ummariense* Strain RL-3, a Hexachlorocyclohexane-Degrading Bacterium

**DOI:** 10.1128/genomeA.00956-13

**Published:** 2013-11-14

**Authors:** Puneet Kohli, Ankita Dua, Naseer Sangwan, Phoebe Oldach, J. P. Khurana, Rup Lal

**Affiliations:** Molecular Biology Laboratory, Department of Zoology, University of Delhi, Delhi, Indiaa; Interdisciplinary Centre for Plant Genomics & Department of Plant Molecular Biology, University of Delhi South Campus, New Delhi, Indiab

## Abstract

Here, we report the draft genome sequence of the hexachlorocyclohexane (HCH)-degrading bacterium *Sphingobium ummariense* strain RL-3, which was isolated from the HCH dumpsite located in Lucknow, India (27°00′N and 81°09′E). The annotated draft genome sequence (4.75 Mb) of strain RL-3 consisted of 139 contigs, 4,645 coding sequences, and 65% G+C content.

## GENOME ANNOUNCEMENT

*Sphingobium ummariense* strain RL-3 was isolated from the hexachlorocyclohexane (HCH) dumpsite created during the purification process of insecticidal γ-HCH isomer from technical HCH (1). The dumpsite is located at Ummari village in Lucknow, India (2). RL-3 has been documented to degrade all four major isomers of HCH, i.e., α-, β-, γ-, and δ-HCH, with a degradation rate comparable to that of *Sphingobium indicum* B90A, the representative avid degrader of HCH (3).

The genome of strain RL-3 was sequenced using Illumina HiSeq 2000 (2 kb paired-end library) and 454 GS FLX titanium platforms. The data were assembled (*n* = 4,706,077; 4.7 Mb) using ABySS assembler version 1.3.3 (4) set at a k-mer length of 57. The assembly generated 139 contigs (*N*_50_ contigs, 363,277 bp), comprising 4,754,053 bp. The final assembly had 80-fold coverage, with the largest contig (GenBank accession number AUWY01000120) of 249,857 bp in size and mean G+C content of 65%.

The draft genome was annotated using RAST version 4.0 (5) and NCBI Prokaryotic Genome Annotation Pipeline (PGAP version 2.1; http://www.ncbi.nlm.nih.gov/genomes/static/Pipeline.html). A total of 4,645 protein-coding sequences and 2,365 hypothetical proteins were predicted. Twelve rRNAs (4 each of 5S, 16S, and 23S), 56 tRNA genes, and 2 pseudogenes were predicted using PGAP.

Out of a battery of *lin* genes that degrade HCH isomers in sphingomonads (6), strain RL-3 was found to contain one copy each of *linA*, *linB*, and *linX* and three copies of *linDER*. Apart from housing the HCH-degradative *lin* genes, strain RL-3 also encodes a diverse array of proteins involved in the degradation of catechol, protocatechuate, and vanillate. Over 50 mobile genetic elements were identified, including IS*6100*, IS*4*, IS*5*, IS*401*, ISsp5, IS*1247*, ISSp1, and IS*1412*. RAST annotations revealed 399 subsystems. *Sphingobium japonicum* UT26S (score, 523), *Sphingomonas* sp. strain SKA58 (score, 484), and *Sphingomonas wittichii* RW1 (score, 424) were identified as the closest neighbors of strain RL-3. Four contigs (accession numbers AUWY01000011, AUWY01000023, AUWY01000029, and AUWY01000134) were assigned (i.e., BLASTN, e-10^-15^) to the reference plasmid genotypes of the HCH degraders *Sphingomonas* sp. strain MM1 and *Sphingobium japonicum* UT26. All this clearly emphasized the role of horizontal gene transfer in shaping the genome of strain RL-3.

We now anticipate that combined analysis of our recent data on sphingomonad genomes (7–13) and metagenomes from the HCH dumpsite (14, 15) coupled with the genome sequence of strain RL-3 presented here may further help our ongoing efforts to elucidate the mechanism of acquisition and evolution of *lin* genes among sphingomonads under HCH pressure. The ability of strain RL-3 to degrade all the major HCH isomers, especially the recalcitrant β-HCH, and the presence of multiple copies of *linDER* as reported in this study further suggest the application of this strain in the development of a consortium for HCH bioremediation.

### Nucleotide sequence accession numbers.

The genome sequence of *Sphingobium ummariense* RL-3 has been assigned the GenBank accession number AUWY00000000. The version described in this paper is version AUWY01000000.
